# Functional Topography of Auditory Areas Derived From the Combination of Electrophysiological Recordings and Cortical Electrical Stimulation

**DOI:** 10.3389/fnhum.2021.702773

**Published:** 2021-08-17

**Authors:** Agnès Trébuchon, F.-Xavier Alario, Catherine Liégeois-Chauvel

**Affiliations:** ^1^INSERM, Institute of Systems Neuroscience, Aix-Marseille University, Marseille, France; ^2^CNRS, LPC, Aix-Marseille University, Marseille, France; ^3^Department of Neurological Surgery, School of Medicine, University of Pittsburgh, Pittsburgh, PA, United States

**Keywords:** cortical stimulation, auditory areas, functional mapping, intracerebral recordings, language

## Abstract

The posterior part of the superior temporal gyrus (STG) has long been known to be a crucial hub for auditory and language processing, at the crossroad of the functionally defined ventral and dorsal pathways. Anatomical studies have shown that this “auditory cortex” is composed of several cytoarchitectonic areas whose limits do not consistently match macro-anatomic landmarks like gyral and sulcal borders. The only method to record and accurately distinguish neuronal activity from the different auditory sub-fields of primary auditory cortex, located in the tip of Heschl and deeply buried in the Sylvian fissure, is to use stereotaxically implanted depth electrodes (Stereo-EEG) for pre-surgical evaluation of patients with epilepsy. In this prospective, we focused on how anatomo-functional delineation in Heschl’s gyrus (HG), Planum Temporale (PT), the posterior part of the STG anterior to HG, the posterior superior temporal sulcus (STS), and the region at the parietal-temporal boundary commonly labeled “SPT” can be achieved using data from electrical cortical stimulation combined with electrophysiological recordings during listening to pure tones and syllables. We show the differences in functional roles between the primary and non-primary auditory areas, in the left and the right hemispheres. We discuss how these findings help understanding the auditory semiology of certain epileptic seizures and, more generally, the neural substrate of hemispheric specialization for language.

## Introduction

Mapping cortical auditory functions in humans has provided valuable insights about inter-areal anatomo-physiological distinctions, or about left-right functional asymmetries. This approach has updated our vision of auditory cortex and of the hemispheric dominance for language. Here, we describe how electrical cortical stimulation can be combined with anatomy and electrophysiology to decipher the sensory and cognitive aspects of the auditory functions.

## Anatomical Description of the Auditory Cortex

**FIGURE 1:**
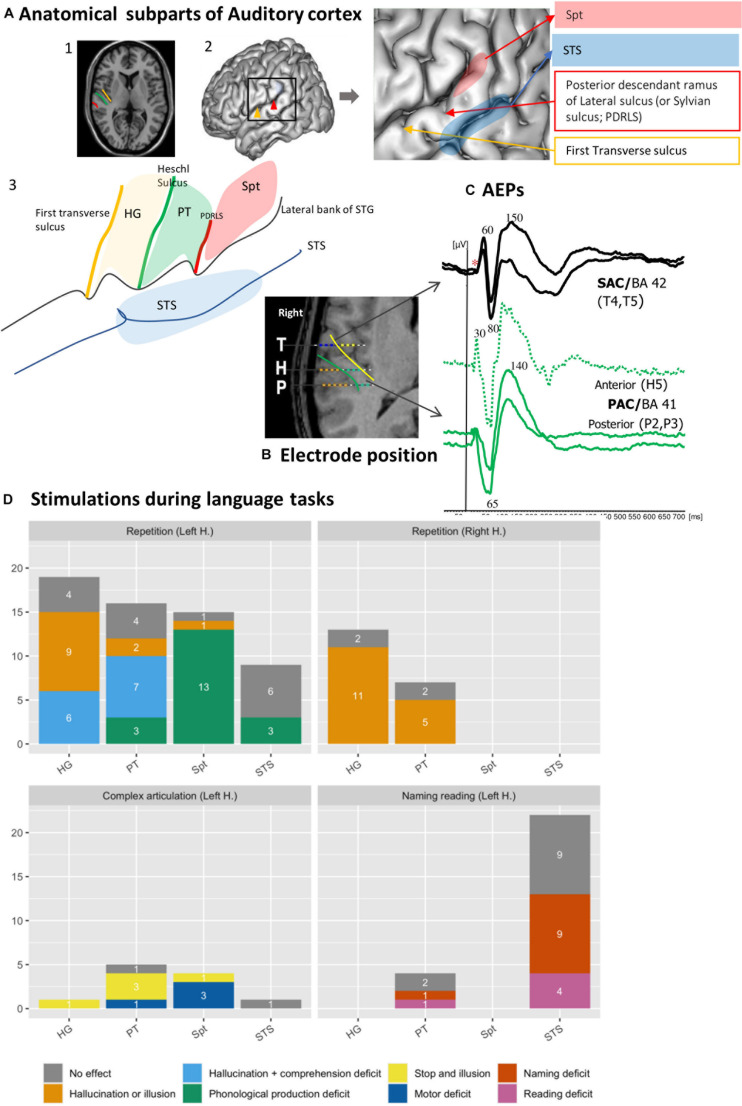
Functional heterogeneity of auditory cortices. **(A)** Anatomical subpart of Auditory Cortices. The subparts of auditory cortices are delimited according to the different sulcus of the posterior part of the temporal Gyrus. Heschl’s Gyrus (HG) by the transverse sulcus (yellow line) and posteriorly by Heschl sulcus (HS, green line). The Planum Temporale (PT) is limited anteriorly by the HS and posteriorly the horizontal PT is limited where the Sylvian sulcus splits into ascending ramus and descending ramus (red line). The Sylvian-Parieto-temporal region (Spt in red) lies between PDRLS and the posterior ascendant ramus of the sylvian sulcus. The several subparts according to the anatomical landmark are showed on (1) MRI axial view of auditory cortex; (2) 3D lateral brain representation and (3) on a schematic representation of auditory regions described above. **(B)** Example of electrode position along the auditory cortex. The 3 electrodes cross the two main sulci. The medial contacts of T electrode (yellow dots), explore the planum polare and the lateral contacts (blue dots) after crossing the transverse sulcus (yellow line) record the anterior part of HG (BA 42). H explore the medial part of HG (green dots, BA 41), then after crossing HS (green line) explore PT. The electrode contacts of P explore the medial part of HG (green dots, BA 41), then after crossing HS (green) explore PT. **(C)** Example of AEPS in the several sub-part of the auditory cortices in response of tone burst sound. Note the difference of the latency of the first component, around 30 ms for primary auditory cortex (BA 41) and 60 ms for secondary auditory cortex. **(D)** Stimulation of auditory cortex according to the subpart of the auditory cortices during language task (original data). The *Y*-axis corresponds to the number of stimulations performed, and the color codes for the different type of errors. Each bars graph corresponds to a sub-region (HG, PT, STS, and Spt). Each panel corresponds to repetition on left or right hemisphere (1 and 2), motor (3) and reading and naming (4) tasks. Solely hallucinations or illusions are predominantly induced in HG and PT and, specifically in the left hemisphere, sometimes accompanied by comprehension deficit. Stimulation of Spt induced phonological errors. The deficit in Naming and reading task is mainly observed in the posterior part of the STS. *Absence of early primary components.

The auditory cortex in humans is largely confined in the posterior part of the superior temporal gyrus (STG), including Heschl’s gyrus (HG) and Planum Temporale (PT) (Figure 1A). The precise posterior and anterior boundaries of these structures within STG have not been clearly defined (Berman et al., 2013), but it is clearly established that this territory is composed of several anatomically and physiologically distinct sub areas (Brodmann, 1909; Braak, 1980; Rivier and Clarke, 1997; Morosan et al., 2001). Once these multiple subdivisions are identified, a consistent pattern can be discerned as follows.

The core region labeled primary cortex (BA 41) is easily identified on the basis of its cytoarchitectonic structure (Braak, 1980). Macroscopically, it appears to be deeply buried in the sylvian fissure, confined to the postero-medial two thirds of HG, with substantial inter-hemispheric and inter-individual anatomical variations (Rademacher et al., 1993; Hackett, 2015). The primary auditory cortex is flanked by bands of secondary areas (BA 42) that extend in the lateral part of HG and, posteriorly, toward the PT. Anteriorly, on to the Planum Polare, lie associative areas (BA 22). Posteriorly, the ascendant segment of the PT has been distinguished from its horizontal segment based on where the Sylvian sulcus splits into ascending ramus and descending ramus, labeled as Sylvian-Parieto-temporal region (Spt) (Witelson and Kigar, 1992; Sweet et al., 2005). This simplified description provides a standard working hypothesis that disregards more elaborate accounts where up to 30 anatomical sub-areas may be distinguished (Galaburda and Sanides, 1980; Wallace et al., 2002; Hackett, 2015).

## Anatomo-Functional Delineation of Auditory Areas

*In vivo* electrophysiological recordings and direct electrical stimulation are invasive experimental methods that can be used to advance our understanding of the human auditory cortex. This type of research is performed in patients with intractable epilepsy undergoing pre-surgical diagnostic investigations with a stereotactic method [stereo-electroencephalography (SEEG)] involving depth electrodes (Chauvel et al., 2019), or with electrocorticographic (ECoG) electrodes apposed on the surface of the brain (Hamberger et al., 2005). The goal of the pre-surgical evaluation protocol is to define the organization of the epileptogenic zone as well as the functionally “eloquent” cortical regions. While ECoG recordings provide surface cortical maps of gyral activity, SEEG electrodes record activity from both gyri and sulci; importantly, SEEG access deep cortical structures, allowing to disentangle activity from sub-regions within auditory cortex (Figure 1B).

### Auditory Evoked Potentials

The latencies of auditory evoked potentials (AEPs) elicited by clicks or pure tones reflect an anatomical segregation (Figure 1C). The sources of the different components lie in HG along the medio-lateral axis. Primary components (latencies below 30 ms) are generated in the tip of HG allowing a physiological delineation of primary auditory cortex (BA 41) (Liégeois-Chauvel et al., 1991). Sources with intermediate latency components (50–80 ms) are distributed from the lateral part of BA 41 to BA 42. Late components (above 80 ms) are generated in BA 42, the lateral parts of HG and PT, and at the posterior part of STG (BA 22) (Celesia and Puletti, 1969; Celesia, 1976; Liégeois-Chauvel et al., 1994, 2003, 2004; Howard et al., 2000; Brugge et al., 2008, 2009).

### Spectro-Temporal Analysis

Time frequency analysis (TFA) has been important for revealing non-phase locked cortical activity and allowed for distinguishing single-trial spatio-temporal response patterns elicited across the auditory cortex by verbal and non-verbal stimulations (Chang et al., 2010). These patterns provide evidence for the tuning properties of cortical sites (Nourski et al., 2014) and they are modulated by the repetition rate of the stimulation (Nourski and Brugge, 2011). All these patterns reveal the representation of stimulus features which can be used to predict responses to novel stimuli or reconstruct the presented stimuli from pattern of cortical activity (Pasley et al., 2012).

### Electrical Stimulation of Auditory Areas

The functional properties of the human auditory cortex were first described by W. Penfield using electrical stimulation to perform functional mapping during awake craniotomy procedures. The primary goal was to generate seizures to localize their origin. The clinical effects of the stimulation of each site were documented, along with intraoperative photographs of the anatomic locations of the stimulated sites. In 1938, Penfield reported hallucinations of sounds previously heard or experienced, provoked by electrical stimulation of the temporal cortex. In subsequent seminal publications, Penfield and collaborators showed that the primary auditory area lied on the anterior part of HG within the sylvian fissure (Penfield and Rasmussen, 1950; Penfield and Jasper, 1954; Mullan and Penfield, 1959). The stimulation of those locations resulted in an auditory sensation like a tone, a buzzing, or knocking sounds. This research thread is summarized in Penfield and Perot (1963).

An anatomo-functional dissociation was proposed between the sites from which electrical stimulation triggered elementary auditory hallucinations (i.e., crude auditory sensations) versus auditory illusions (i.e., altered interpretations of heard sounds: “sounds heard seemed louder or clearer, fainter or more distinct, nearer or farther”). The auditory hallucinations were triggered by the stimulation of the deep part of HG while the illusions were linked to the lateral part of HG, extending forward and back along the STG. More complex auditory “psychical responses” (e.g., relatives’ voices, music, and meaningful sounds) have been reported mostly after stimulation of the Planum Polare. They probably result from a complex and widely distributed activation, involving brain regions beyond the auditory cortex.

In De Graaf et al.’s (2000) study, most of stimulation (62% of 180 stimulations) provoked auditory subjective symptoms. Among them, 32% consisted in simple hallucinations, mainly recorded in the postero-medial part of HG (BA 41). On the contrary, stimulation of the lateral part of HG provoked more illusions than hallucinations. More generally, there was a gradient in the subjective responses from area 41 to 42 in HG, changing from high frequency sounds to broadband noise, to illusions. In the PT, auditory illusions and hallucinations were observed with equal frequency. Anteriorly to HG (BA 22), illusions were most often reported. They could be perceived contralateral to the stimulation or bilaterally. In summary, stimulation data consistently reveal two types of positive responses, with a clear-cut difference in the subjective auditory symptoms between the stimulation of BA 41 (primary cortex) eliciting mostly hallucinations and BA 42’s (secondary cortex) provoking illusions. This is in line with the functional differences in electrophysiological responses (early vs. mid latency evoked components, respectively) recorded from these areas. The stimulation of the posterior lateral superior temporal area at the site of maximal potentials evoked by clicks elicited either hallucinations or illusions (Howard et al., 2000).

More rarely, hearing suppression was observed, for example following the stimulation of the “posterolateral aspect of the STG” or the “anterior part of HG” (Mullan and Penfield, 1959) see also (Sinha et al., 2005) and (Fenoy et al., 2006). Those hearing suppressions were not lateralized and they outlasted the duration of the stimulation. They could be accompanied by an altered perception of the timing in series of acoustic stimuli, or by a temporal dissociation between the experimenter’s lips movements and the speech sounds they uttered.

### Effects on Language Processing

Besides inducing auditory sensations, electrical cortical stimulations can impair language perception and production when they are delivered during behavioral tasks (Trébuchon and Chauvel, 2016). Figure 1D shows the outcome of 117 stimulations of 39 electrodes from 26 patients, sorted according to the different sub-regions of posterior STG. During a word repetition task, left HG and PT stimulation produced hallucinations or illusions sometimes along with comprehension deficit. Articulatory or phonological errors are elicited by the stimulation of Spt during word or pseudo word repetition, presumably due to a difficulty to maintain task-relevant representations in a phonological loop [in keeping with (Buchsbaum et al., 2011; Hickok, 2012)]. Lastly, the posterior part of left STS seems involved in more high-level language processes required in naming and reading tasks, because its stimulation did not induce positive auditory symptoms but naming or reading deficits (e.g., delayed responses, phonological errors, or semantics errors). The reading deficit included grapheme decoding, comprehension deficit and grapheme to phoneme deficit.

The contrastive consequences HG and PT electrical stimulations have been replicated in a recent study where stimulations were applied at the onset or the offset of a sentence the patient was asked to repeat (Forseth et al., 2020). Speech comprehension was disrupted by the stimulation of HG at the onset of the sentence while the disruption of speech production was observed when the stimulation of PT was applied at the end of the sentence, about the time when the patient must start to repeat. Performance impairments were observed specifically in the left hemisphere in cases of typical language organization. When we compared left/right HG and PT stimulations during a repetition task we did not observe comprehension deficits on the right side (Figure 1D, bottom panel). The fact that comprehension deficits are selectively observed in the language specialized hemisphere is consistent with the hypothesis that the hemispheric dominance would result from the asymmetry of auditory cortical tuning (Liégeois-Chauvel et al., 1999; Kell et al., 2011).

## Functional Asymmetry Between the Right and Left Auditory Cortices

There is suggestive neuroanatomical evidence for structural differences between the left and right auditory cortices in humans. The primary auditory cortex (BA 41) is larger in the left hemisphere, with a higher density of gray and white matter, irrespective of handedness (Penhune et al., 1996; Dorsaint-Pierre et al., 2006). The left auditory cortex (HG and PT) contains larger cortical columns than its right counterpart, with a higher number of large pyramidal cells in cortical layer III (Hutsler and Galuske, 2003). The PT, or secondary auditory cortex, is also larger in the left hemisphere in the majority of individuals, and this structural asymmetry is related with the hemispheric dominance for language (Shapleske et al., 1999). Such differences in cytoarchitectonic organization coincide with electrophysiological and functional differences between auditory regions.

Building on these observations, Poeppel (2003) hypothesized that two endogenous oscillations, in the low-gamma (25–45 Hz) and in the theta (4–8 Hz) bands, underlie asymmetric sampling in time (AST) of auditory signals. These two rhythms are asymmetric at rest in HG, with theta dominating in right and gamma in left auditory cortex (Sinha et al., 2005; Fenoy et al., 2006). This observation is compatible with distinct integration properties in right and left auditory cortices underlying the chunking of continuous speech into phonemic and syllabic segments, respectively, (Poeppel, 2003; Giraud and Poeppel, 2012). This functional asymmetry is a plausible neurophysiological substrate of the greater sensitivity of the left auditory cortex to short sound segments and brief speech features (Jamison et al., 2006; Obleser et al., 2008) and of the greater sensitivity of the right auditory cortex to slower acoustic fluctuations and longer steady speech signals such as vowels and syllables (Boemio et al., 2005; Abrams et al., 2008).

As a paradigmatic example, consider Voice Onset Time (VOT), which is the primary phonetic cue for the phonological distinction between voiced and voiceless stop consonants in a large variety of languages (Serniclaes, 1987; Cho and Ladefoged, 1999). VOT is the time lag between the release of the oral constriction for the consonant and the onset of the vibration of the vocal folds [i.e., the voicing: (Lisker and Abramson, 1964)].

**FIGURE 2:**
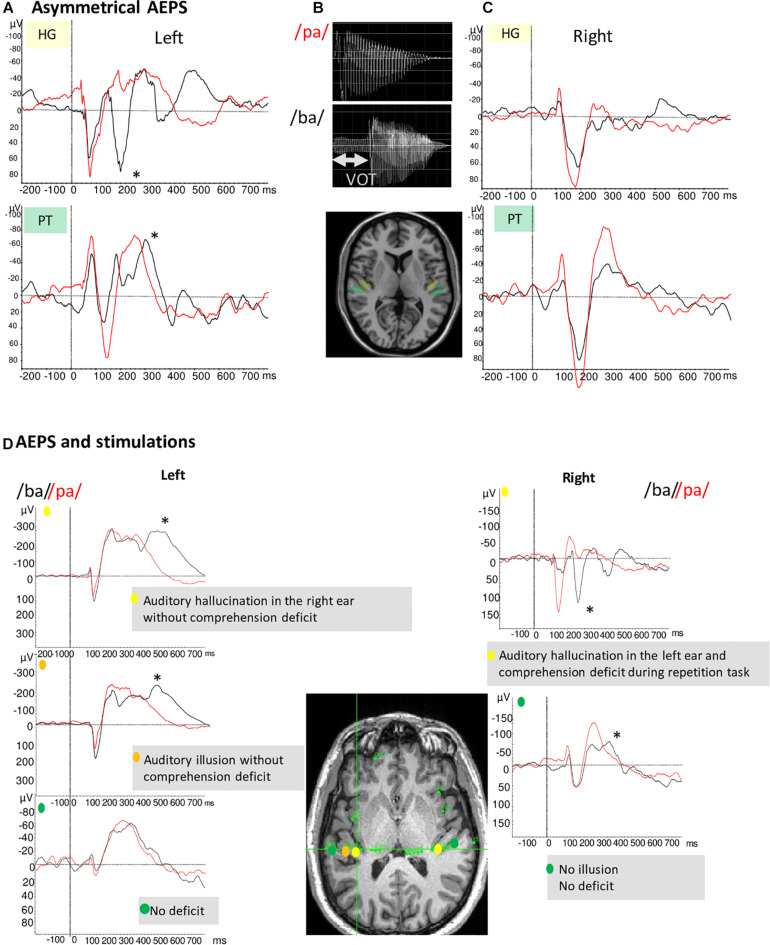
**(A,C)** Illustration of example of AEP in response to voice /ba/ and /pa/ of two patients with typical language organization (according to handedness: RH, fMRI-activation in the left hemisphere during language task, stimulation, and ictal speech disturbance in the left hemisphere). **(A)** The temporal auditory coding of VOT took place specifically in the left HG and PT **(A)**. The /ba/ (black curve) elicited a first complex N1/P2 at the onset of voicing and a second component (marked by *) time-locked to release whereas the /pa/(red curve) elicited only one complex N1/P2. **(C)** On the right PT and HG no difference between /pa/ and /ba/ is observed. **(B)** Voiced stop consonants /ba/ and voiceless stop consonants /pa/. The VOT refers to the time between a phonetically relevant supra-laryngeal event, such as release, and glottal paulsing. In French, VOT it is a long negative value (∼ –110 ms) for voiced stop consonants (/ba/) and a short positive value (∼ +20 ms) for voiceless syllable (/pa/). **(D)** AEP in response to voice /ba/ and /pa/ and stimulations results of one patient with atypical language organization (left handed; no language deficit during seizure and stimulation of the left temporal regions whereas stimulation of the right temporal region induced language deficit). AEP and stimulations results are presented together to underline their complementarity. Left hemisphere/AEP, the temporal auditory coding of VOT took place in the left HG; Left hemisphere/Stimulation, typical hallucination and illusion are elicited in two parts of HG; Right hemisphere/AEP, the temporal auditory coding of VOT has been recorded on the HG and the PT; Right hemisphere/Stimulation, the stimulation of the right HG induced auditory hallucinations associated to a comprehension deficit.

Several studies have reported VOT discrimination deficits in patients with damage to the left hemisphere (Blumstein et al., 1977). Liégeois-Chauvel et al. (1999) showed lateralized processing of acoustic elements of the French voiced stops (e.g., /ba/) by time locking neural signals in the left dominant auditory cortex to the consonant onset or the release burst. These findings have been replicated and used as an electrophysiological marker of the hemispheric dominance for language (Trébuchon et al., 2005). Figures 2A–C illustrates the asymmetry between left and right auditory cortices in the case of a typical left organization for language. Conversely, Figure 2D (bottom frame) shows one patient with an atypical language organization. These temporal processing patterns are a function of the specific features of the syllables, with different electrophysiological patterns across languages (e.g., English vs. French). However, regardless the native language of the patients, the enhanced sensitivity to the temporal acoustic characteristics of sounds that is only present in BA 41 and BA 42 reflects information processes needed for tagging further phonetic processing which likely take place in BA 22 (Morillon et al., 2012; Giroud et al., 2020).

Following spectro-temporal analysis of the acoustic signal in the auditory cortex which is the first stage of the speech processing, the phonetic and phonological processes take place in posterior part of STG (Hickok and Poeppel, 2007; Morillon et al., 2012). High gamma frequency band has been correlated with phonetic and categorical features (Chang et al., 2010). More recently, decoding algorithms have been developed, synthetizing acoustic features from parameters predicted from the brain activity. These studies showed successful decoding of spectro-temporal features of speech in the STG [for review see, (Martin, 2019)].

### Ictal Auditory Symptomatology

Auditory auras reported by patients during spontaneous seizures include a spectrum of phenomena ranging from simple auditory hallucinations to complex hallucinations or illusions. Simple auditory hallucinations, when they occur as a first ictal sign, are reliable signs to localize the epileptogenic zone (EZ) in the lateral posterior temporal regions (Maillard et al., 2004; Barba et al., 2007). Auditory hallucinations are most often heard contra-laterally to the EZ, which is largely consistent with what is observed during electrical cortical stimulations. Retrospective studies including a large number of patients show that the prevalence is weak (∼2% of the temporal lobe patients) (Florindo et al., 2006; Balgetir et al., 2018). The localization value of illusion is less consistent and suggest a more large and complex organization of the EZ, for instance in case of temporal lobe plus epilepsy (Barba et al., 2007). Auditory auras has been also report in context of autosomal dominant partial epilepsy characterized by auditory features (Ottman et al., 1995) for which the responsible gene LGI1 has been defined (Morante-Redolat, 2002).

There are only a few case reports of ictal verbal and musical hallucinations. Verbal hallucinations appear to be linked to EZs in the dominant hemisphere (Florindo et al., 2006) while musical hallucinations are linked to the right temporal lobe regardless of dominance (Wieser, 1980; Fénelon et al., 1993; Griffiths et al., 1997) [reviewed in Wieser et al. (1997), Kaplan (2003)]. As was argued for stimulation-induced hallucinations of similar content, hallucinatory perceptions may be construed as re-experiencing stored perceptual experiences, presumably involving a broad network.

Finally, ictal illusions or post-ictal palinacousis (i.e., auditory illusions consisting of the perseveration or echoing of an external auditory stimulus after it has ceased) are rarely reported in patients with temporo-parietal seizures [for a review, (Di Dio et al., 2007)].

## Applications to Tinnitus

Auditory hallucinations evoked by electrical cortical stimulation share features with tinnitus, commonly defined as the perception of sound in the absence of an external auditory source. The rare observations of hearing suppression after cortical stimulation have opened new perspectives to treat tinnitus. It has been postulated in tinnitus patients that networks connecting the primary sensory cortices to other cortical areas and the periphery exhibit hyperexcitability leading to hallucinations (De Ridder et al., 2014).

In this context, a treatment strategy which seems to generate long and robust tinnitus suppression is to stimulate the auditory cortex using epidural electrodes (De Ridder et al., 2006, 2011).

The seemingly contradictory behavioral effects of suppression in tinnitus vs. hallucinations-illusions in epileptic patients could depend on the stimulation parameters and on the individual state of the cortical sites. In tinnitus, the peripheral deafferentation (hair cell deterioration) changes the spontaneous rate, synchrony and entails a cortical reorganization; the electrical stimulation might induce a decorrelation of the spontaneous activity. On the healthy auditory cortex, stimulations excite all neurons and elicit hallucinations (as noted above, only in rare cases does it result in temporary deafness).

## Discussion: Interpreting the Symptomatology Elicited by Electrical Stimulation for Clinical and Research Purposes

It is important to remember that electrical stimulation during SEEG explorations is performed primarily for eliciting seizures. The electrophysiological mapping of sensory and associative areas involved in cognitive networks should be conducted alongside, to answer the fundamental question of whether there is a spatio-temporal overlap between the epileptogenic and the functional networks. The identification of cortical structures that are essential to cognitive or perceptual functions is challenging because the human brain is a complex system in which a vast range of function arises from coordinated neural activity across diverse spatial and temporal scales (Sporns and Betzel, 2016; Bassett and Bullmore, 2017).

Effects arising from the stimulation of the primary sensory cortices are more localizing than that of associative cortices which involves the activation of a network or networks that underpins the functional emergence of language impairments.

Trebuchon (Trébuchon, 2021) described the procedure to follow, including stimulation parameters such as duration and timing, to avoid pitfalls such as “false negative stimulation.” They also described how to interpret the symptoms in relation with collateral electrophysiological changes such as after-discharges. It is especially important to interpret the role of the PT and the posterior part of the superior temporal sulcus in language perception and production. Choosing the task according to the stimulated sub-region is particularly crucial (Figure 1A). HG and PT should be tested with a repetition or repetition and designation task; Spt should be tested with repetition and repetitive motor tasks, STS should be preferentially tested with naming and reading tasks.

The relationship between phenomena induced by cortical electrical stimulation and normal brain physiology is also a fair question to ask, given that the epilepsy condition may result in the functional alterations of the networks it affects. The auditory manifestations following the stimulation of auditory cortex could result from a perturbation of the efferent pathway between the cortex and the periphery (cochlea), that would lead to abnormal auditory processing.

A stimulation induced deficit could result from an inhibitory effect of the stimulation due to the temporary inactivation of a local population of neurons, either pyramidal cells or interneurons. However, we are far from a perfect understanding of the functional or physiological effects of pulse or train stimulations. It is very common that the stimulation of the same region with the same parameters leads to various effects. One explanation could be that the “inhibitory effect” of the stimulation induces a rapid plasticity of the system that lasts for at least a few minutes following the trial (Trébuchon, 2021).

Overall, the available data favor the view that a positive response is evidence of an activation of the stimulated cortical neurons while a negative response could be interpreted as an inhibition of behavior attributed to neuronal inactivation (Borchers et al., 2012). The stimulation of auditory cortex at the base of the cortical hierarchy of networks involved in auditory perception elicited frequent and simple effects and allows a reliable assessment of sensory function. But these effects become increasingly rare, heterogeneous and complex in heteromodal networks making the evaluation of speech perception and production functions more uncertain (Fox et al., 2020).

The use of single pulse electrical stimulation could help to resolve how adjacent and remote areas are inter-connected by measuring the cortico-cortical evoked potentials and identify the role of the auditory cortex in the language network [(Matsumoto et al., 2004, 2017), for review see David et al. (2010)].

In our view, solid physiological foundations underlying the effect of electrical stimulation need to be established, and the labeling of direct electrical stimulation as the “gold standard for mapping brain function” remains the matter of an interesting debate (Grande et al., 2020).

## Data Availability Statement

The original contributions presented in the study are included in the article/supplementary material, further inquiries can be directed to the corresponding author.

## Ethics Statement

The studies involving human participants were reviewed and approved by the Comite de Protection des Personnes. The patients/participants provided their written informed consent to participate in this study.

## Author Contributions

All authors listed have made a substantial, direct and intellectual contribution to the work, and approved it for publication.

## Conflict of Interest

The authors declare that the research was conducted in the absence of any commercial or financial relationships that could be construed as a potential conflict of interest.

## Publisher’s Note

All claims expressed in this article are solely those of the authors and do not necessarily represent those of their affiliated organizations, or those of the publisher, the editors and the reviewers. Any product that may be evaluated in this article, or claim that may be made by its manufacturer, is not guaranteed or endorsed by the publisher.
